# Book Review

**Published:** 1948

**Authors:** 


### Reviews of Books

**Emergency Surgery :** Part I. By HAMILTON BAILEY, F.R.C.S., F.A.C.S., F.I.C.S., F.R.S.E. Sixth Edition. Illustrated. Pp. viii, 180. Bristol: John Wright & Sons Ltd. 1948. Price 21s. net.—Hamilton Bailey's *Emergency Surgery* (Part I) contains all the up-to-date information on the subject. It is rare to find an author with so fervent and trenchant a way of telling the surgical story. The newer procedures for transfusion of blood, etc., and the dangers from their abuse by the unwary are fully described and the simple safe Baxter type of infusion depicted. Anaesthesia, its risks and their remedies, are handled comprehensively. Abdominal trauma, appendicitis and other hypogastric sources of peritonitis are all well explained and afford a copiously illustrated guide for the younger surgeons. It is essentially a practical text-book and describes with meticulous detail the latest technical manoeuvres and explains their rationale. The author's style is appropriately incisive and forthright throughout, and it is obvious that he writes from the daily experience of one who is constantly at the " Surgical Bench". While nothing can replace the influence of experience in emergency surgery, it is from guides such as this that the young surgeon is inspired to aim at the highest standard of service.

**Emergency Surgery:** Part II. By HAMILTON BAILEY, F.R.C.S., F.A.C.S., F.I.C.S., F.R.S.E. Sixth Edition. Pp. 181-388. Illustrated. Bristol : John Wright & Sons Ltd. 1948. Price 21s.—The first subject in this volume is fittingly one of the most beneficent chapters in emergency surgery—the closure of perforated ulcers—with its sections on first-aid, mortality charts, new devices for dealing with difficult cases. One can want no better exposition on this catastrophe commonly overtaking busy men in the prime of life. All Britain needs is beds and doctors to give effect to such first-class instruction. In the biliary sphere, immediate operation might be emphasized in cases where the clinician senses virulent trouble but finds no decisive signs regarding pathology. Intervention in the incipient stage alone may disclose dire alternative diagnoses, such as sub-hepatic appendix, perforated ulcer or bowel. Operating promptly may therefore make all the difference, and need do no harm in the ordinary biliary case. The rarer casualties —swallowed foreign bodies and trauma—are handled in a lucid and competent manner. The page on obstruction after acute appendicitis is a model of practical wisdom in a most anxious situation. The patient, often youthful, a week or so later is suddenly at death's door. It is a tense situation from which he can only emerge safely and the initial surgical triumph be consummated by prompt second operation. Dealing with bowel obstruction due to gallstone, digital disintegration receives no mention. Admirable pictures and diagrams clarify the chapter on strangulated hernia. Dogmatic generalizations appear to which exception might be taken, and the page of sketches on biliary surgery is more picturesque than practical, but this liberally-illustrated book is a shining spearhead in the vanguard of modern surgery.

**The Care of Tuberculosis in the Home.** By JAMES MAXWELL, M.D., F.R.C.P. Second Edition. Pp. xii, 113. Illustrated. London: Hodder & Stoughton. 1947. Price 7s. 6d.—At once there looms before the individual confronted with a diagnosis of pulmonary tuberculosis a broad vista of urgent problems, both for himself and his family. If he is to gain the maximum benefit in health, either in his home or in sanatorium, the most vital problem is to make him acquainted, as rapidly as possible, with the nature of the disease, the treatment required, the dangers of infection and the necessary modifications of his future life. This book of 112 pages is written for the patient with this object in view, and it can be stated that the object is successfully achieved. Technical language has been avoided; the book is eminently readable and understandable. Some adverse criticisms can, of course, be found—for example, diagrams should be added; in discussing contacts the importance of X-rays could have been stressed and kissing deprecated. Umckaloabo is unworthy of a whole page, while streptomycin, para-aminosalicylic acid and B.C.G. vaccine receive no mention—and patients like to be up to date! Drawbacks to thoracoplasty are overrated, and the "acquisition of tuberculosis infection by the great majority of children within the first five years of life" is a highly questionable statement. It should be stated that milk is rendered safe by boiling. Resettlement and home crafts would have been welcomed. The price, 7s. 6d., may tend to limit the widest distribution which this book deserves.

**Social Services in Bristol 1948.** Compiled by the BRISTOL COUNCIL OF SOCIAL SERVICE. Pp. xx, 96. Bristol: J. W. Arrowsmith, Ltd. 1948. Price 3s.—This little book provides in convenient form a complete guide to the city's social services. Beginning with a list of reference books, national and local, it continues with a summary of statutory services, and addresses of Government Departments, arranged in the same way. The largest part of the book is devoted to an alphabetical list of voluntary agencies, their objects, activities, and addresses. Another large section gives appendices on hospitals, homes, almshouses, clubs, and various societies. There are a very complete index and notes on " what to do in urgent cases". It is quite indispensable for all those active or interested in the social work of the city.

